# Horner's Syndrome Incidental to Medullary Thyroid Carcinoma Excision: Case Report and Brief Literature Review

**DOI:** 10.1155/2016/7348175

**Published:** 2016-04-21

**Authors:** Nicholas S. Mastronikolis, Sofia P. Spiliopoulou, Vassiliki Zolota, Theodoros A. Papadas

**Affiliations:** ^1^Department of Otorhinolaryngology, Head and Neck Surgery, University Hospital of Patras Medical School, 26504 Rio, Patras, Greece; ^2^Department of Pathology, University Hospital of Patras Medical School, 26504 Rio, Patras, Greece

## Abstract

Horner's syndrome is characterized by a combination of ipsilateral miosis, blepharoptosis, enophthalmos, facial anhidrosis, and iris heterochromia in existence of congenital lesions. The syndrome results from a disruption of the ipsilateral sympathetic innervation of the eye and ocular adnexa at different levels. Though rare, thyroid and neck surgery could be considered as possible causes of this clinical entity. We present a case of Horner's syndrome in a patient after total thyroidectomy and neck dissection for medullary thyroid cancer with neck nodal disease and attempt a brief review of the relevant literature.

## 1. Introduction

Claude Bernard, a French physiologist, in 1852, first carried out a physiological description of the cervical sympathetic innervation in animals and Johann Friedrich Horner, a Swiss ophthalmologist, first described in 1869 the so-called Claude Bernard-Horner's syndrome (oculosympathetic paresis) in humans [1]. The syndrome is characterized by ipsilateral miosis, blepharoptosis, enophthalmos, facial anhidrosis, and, occasionally, iris heterochromia when congenital lesions exist [2]. It is resulting from a disruption of the ipsilateral sympathetic innervation of the eye and ocular adnexa at different levels: central, preganglionic, and postganglionic [3]. Central causes include stroke of the posteroinferior cerebellar artery, usually accompanied by other neurological symptoms such as in Wallenberg syndrome. Trauma and tumors are the most common preganglionic lesions of Horner's syndrome [4], while cluster migraine [5] and carotid dissection [6] consist of the most common postganglionic causes. Iatrogenic trauma can occur during various cervical surgical procedures, that is, neck dissection, thyroid, and parathyroid operations [7].

We report a case of Horner's syndrome in a patient who underwent total thyroidectomy and neck dissection and attempt a brief review of the relevant literature.

## 2. Case Report

A 73-year-old woman referred to our service from the endocrinology department suffering from medullary thyroid cancer with cervical metastases. The patient presented a 3 cm palpable, firm mass on the right lobe of the thyroid gland. Ultrasonography (U/S) and computerized tomography (CT) revealed palpable lymph nodes in the right cervical area at level III and a right paratracheal mass displacing but without invading the carotid artery, the esophagus, and the trachea. Thyroid function tests gave normal results. Fine-needle aspiration biopsy (FNAB) of the nodule was consistent with the diagnosis of medullary carcinoma.

A total thyroidectomy was performed along with a modified neck dissection type III at the right side and an elective dissection at levels II, III, and IV at the left side of the neck. The thyroid gland was very hard to palpation and strongly attached to the trachea and carotid sheath, without however invading the posterolateral side of the esophagus. Both recurrent laryngeal nerves were identified and respected and their function was appropriately monitored. Intraoperatively, the patient suffered from bradycardia secondary to carotid manipulation and managed with atropine administration.

**Figure 1:**
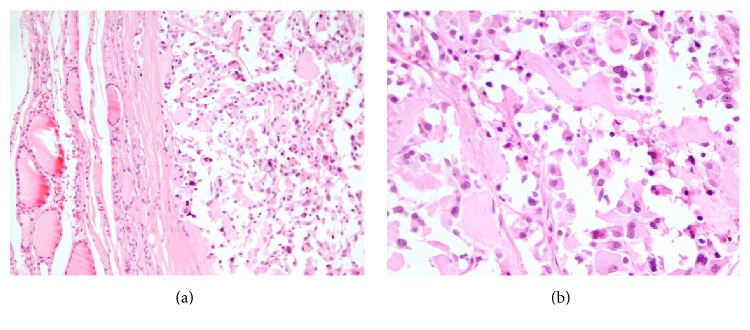
Medullary carcinoma (H + E): nests or chords of cells penetrating dense pink stroma ((a) ×200). Tumor cells were round, relatively uniform with a punctuate chromatin ((b) ×400).

**Figure 2:**
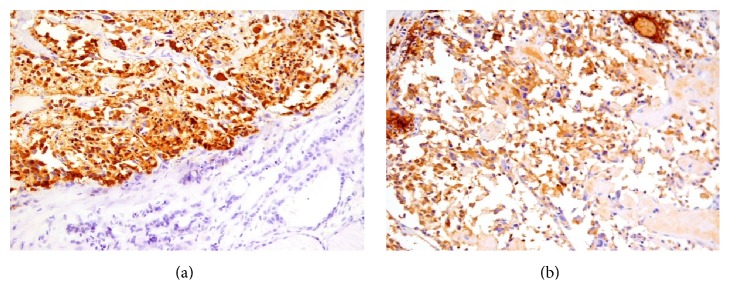
On immunohistochemistry tumor cells were positive for calcitonin ((a) ×200) and CEA ((b) ×200).

Pathological examination of the thyroid gland revealed a 4.3 cm well circumscribed, tan-white, indurated lesion with gritty consistency. The rest of the gland was orange-yellow and fleshy, with no evidence of noted nodules. Microscopy revealed the histological features of medullary carcinoma, such as nests or chords of cells penetrating dense pink stroma with a lobular or trabecular growth pattern (Figure 1(a)). Calcification areas were also noted. On higher power examination, the neoplastic cells were round, relatively uniform with a punctuate chromatin (Figure 1(b)). On immunohistochemistry tumor cells were positive for calcitonin (Figure 2(a)) and CEA (Figure 2(b)).

Four out of thirty-two lymph nodes, found within the tissue removed during neck dissection procedure from the right side, showed metastatic disease.

**Figure 3:**
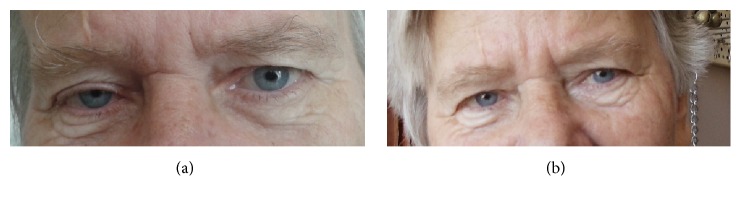
Patient with miosis and blepharoptosis one day after surgery (a). Restoration was achieved four weeks after operation (b).

In the first postoperative day, the patient presented right blepharoptosis (Müller muscle) and miosis (stimuli were slower in the affected pupil) typical of Horner's syndrome (Figure 3(a)). No hematoma, seroma, or infection could be detected as possible cause of the syndrome. The patient exhibited voice weakness (hoarseness) and after laryngeal endoscopy, a palsy of the right vocal cord to the paramedian position was detected due to a potential injury in the communication between the cervical sympathetic chain and the recurrent laryngeal nerve on the right side. No other complication presented in the postoperative period and the patient was discharged from the hospital seven days later. Ptosis and miosis disappeared after four weeks (Figure 3(b)). Stroboscopic examination of the larynx two months after operation revealed restoration of the laryngeal mobility.

## 3. Discussion

Horner's syndrome can rarely occur after neck surgery. Carotid endarterectomy [6] and cervical spine surgery [8] through an anterior approach are considered among the most common causes. Other causes include thyroid and parathyroid surgery [9], excision of a cervical schwannoma [10], ganglioneuroma or paraganglioma, cervical lymph node dissection, drainage of a retropharyngeal or parapharyngeal abscess, and sympathectomy [7]. The cervical sympathetic chain (CSC) is positioned posteromedially to the carotid sheath, anterior to the longus muscle and under the prevertebral fascia, while the cervical sympathetic trunk (CST) can pass within the posterior wall of the carotid sheath. Thus, the chain or the trunk could be directly damaged (a) at the prevertebral fascia during cervical spine surgery, (b) at the paratracheal area during thyroid and parathyroid surgery, and (c) posteromedially at the carotid sheath during the removal of either carotid body tumors, carotid endarterectomy, or other carotid artery procedures. Additionally, injury of the stellate or cervicothoracic ganglion could result from a tube thoracostomy [11] or coronary bypass surgery [12].

According to Cozzaglio et al. [9], Horner's syndrome secondary to thyroidectomy has an incidence of 0.2% after conventional surgery. It can occur directly by mechanical stress, indirectly via an injury of the anastomosis of various nerves and branches following the inferior thyroid artery, or by inflammation and hematoma of this area due to an excessive traction from a retractor. It is notable that the intermediate ganglion of CSC, the smallest one of the three ganglia that compose the chain, is in close proximity with the inferior thyroid artery crossing it anteriorly or posteriorly. Thus, a ganglion injury could happen during the ligation of the inferior thyroid artery producing Horner's syndrome.

In our case, the patient underwent total thyroidectomy, modified radical neck dissection type III in the right side, and elective nodal dissection of levels II–IV in the left side. Horner's syndrome presented in the right side and completely resolved after four weeks. We believe that there was a direct mechanism with traction and trauma of the nerve fibers during retraction of the carotid sheath and possibly an indirect one involving the lesion, mainly the nodal metastasis located at levels IIa/III and VI in the right side.

The prognosis of Horner's syndrome depends on the damage mechanism; in cases of indirect injury, a spontaneous recovery usually takes place, but if there is a complete section, the symptoms will continue.

Although Horner's syndrome is a rare complication, the surgeon must be aware that virtually any cervical surgery, and especially thyroid surgery, may be a source of iatrogenic Horner's syndrome.

A comprehensive knowledge of the anatomy of the CSC and its possible anastomoses with adjacent nerves and cautious surgical dissection, avoiding unnecessary tension with surgical retractors, are crucial to prevent a serious injury.
